# Supplementary material for: Transcriptome analyses describe the consequences of persistent HIF-1 over-activation in Caenorhabditis elegans
Source: bioRxiv. 2023 Nov 17:2023.11.15.567311. Preprint. [Version 1] doi: 10.1101/2023.11.15.567311 (PMC10680707; doi:10.1101/2023.11.15.567311)
Supplement: Supplement 1 [file NIHPP2023.11.15.567311v1-supplement-1.pdf]

# Supporting information

**S1 Table. Gene expression for all the probesets under hypoxia and in the HIF-1 negative regulator mutants.**

**S2 Table. Genes differentially expressed in *vhl-1*, *swan-1;vhl-1*, *egl-9* or *rhy-1*.**

**S3 Table. Overlaps between differentially expressed genes (DEGs) identified by RNA-seq and microarray.**

**S4 Table. Genes up-regulated in *swan-1;vhl-1*.**

**S5 Table. Genes up-regulated in *egl-9*.**

**S6 Table. Genes up-regulated in *rhy-1*.**

**S7 Table. Genes down-regulated in *swan-1;vhl-1*.**

**S8 Table. Genes down-regulated in *egl-9*.**

**S9 Table. Genes down-regulated in *rhy-1*.**

**S10 Table. Genes commonly up-regulated in *swan-1;vhl-1*, *egl-9* and *rhy-1*.**

**S11 Table. Genes commonly down-regulated in *swan-1;vhl-1*, *egl-9* and *rhy-1*.**

**S12 Table. Genes co-upregulated by PMK-1 and HIF-1.**

**S13 Table. Genes co-upregulated by SEK-1 and HIF-1.**

**S14 Table. Genes co-upregulated by Cry5B and HIF-1.**

**S15 Table. Genes co-upregulated by *Yersinia pestis* and HIF-1.**
